# Supplementary material for: Altered Protein Kinase A-Dependent Phosphorylation of Cav1.2 in Left Ventricular Myocardium from Cacna1c Haploinsufficient Rat Hearts
Source: Int J Mol Sci. 2024 Dec 22;25(24):13713. doi: 10.3390/ijms252413713 (PMC11678006; doi:10.3390/ijms252413713)

Supplementary Material

Altered Protein Kinase A-Dependent Phosphorylation of Cav1.2 in Left Ventricular Myocardium from *Cacna1c* Haploinsufficient Rat Hearts

David Königstein [1], Hauke Fender [1], Jelena Plačkić [1], Theresa M. Kisko [2,3,4,5], Markus Wöhr [2,3,4,5] and Jens Kockskämper [1,*]

[1] Institute of Pharmacology and Clinical Pharmacy, Faculty of Pharmacy,
Biochemical and Pharmacological Center (BPC) Marburg, University of Marburg, Germany
[2] Center for Mind, Brain and Behavior (CMBB), University of Marburg, Germany
[3] Behavioral Neuroscience, Experimental and Biological Psychology, University of Marburg, Germany
[4] KU Leuven, Faculty of Psychology and Educational Sciences, Research Unit Brain and Cognition,
Laboratory of Biological Psychology, Social and Affective Neuroscience Research Group, Belgium
[5] KU Leuven, Leuven Brain Institute, Belgium
* Correspondence: jens.kockskaemper@staff.uni-marburg.de; Tel.: +49-6421-2825336

1. Supplementary Western Blot images

1.1 Original Western Blot images from Figure 1

Cav1.3 (from left to right: 4 wildtype samples and 4 *Cacna1c*$^{+/-}$ samples from 8 different animals on each blot, two blots. Membranes were cut before antibody application. Upper membrane contains samples #1-4 of each genotype, lower membrane samples #5-8 of each genotype.)

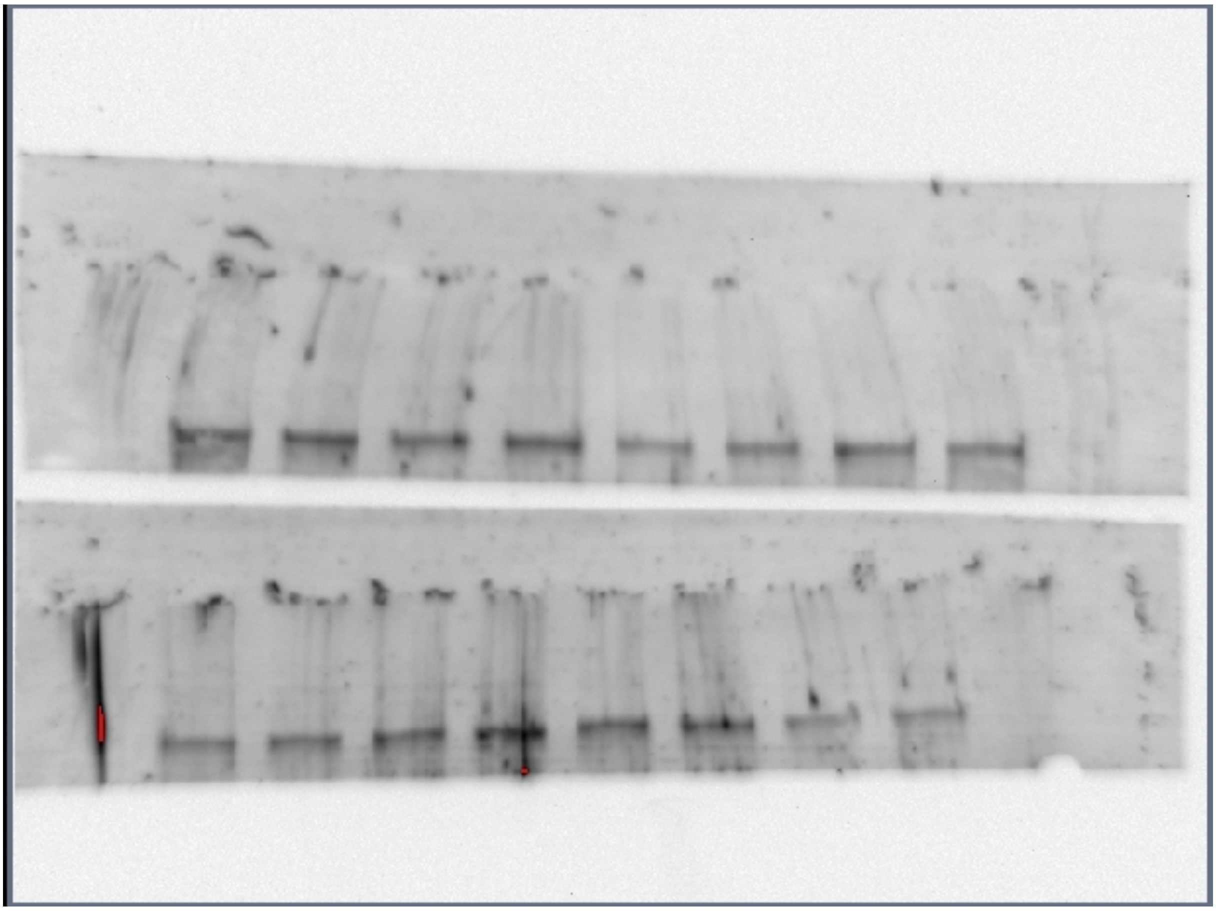

Associated GAPDH membranes (Upper membrane contains samples #1-4 of each genotype, lower membrane samples #5-8 of each genotype.)

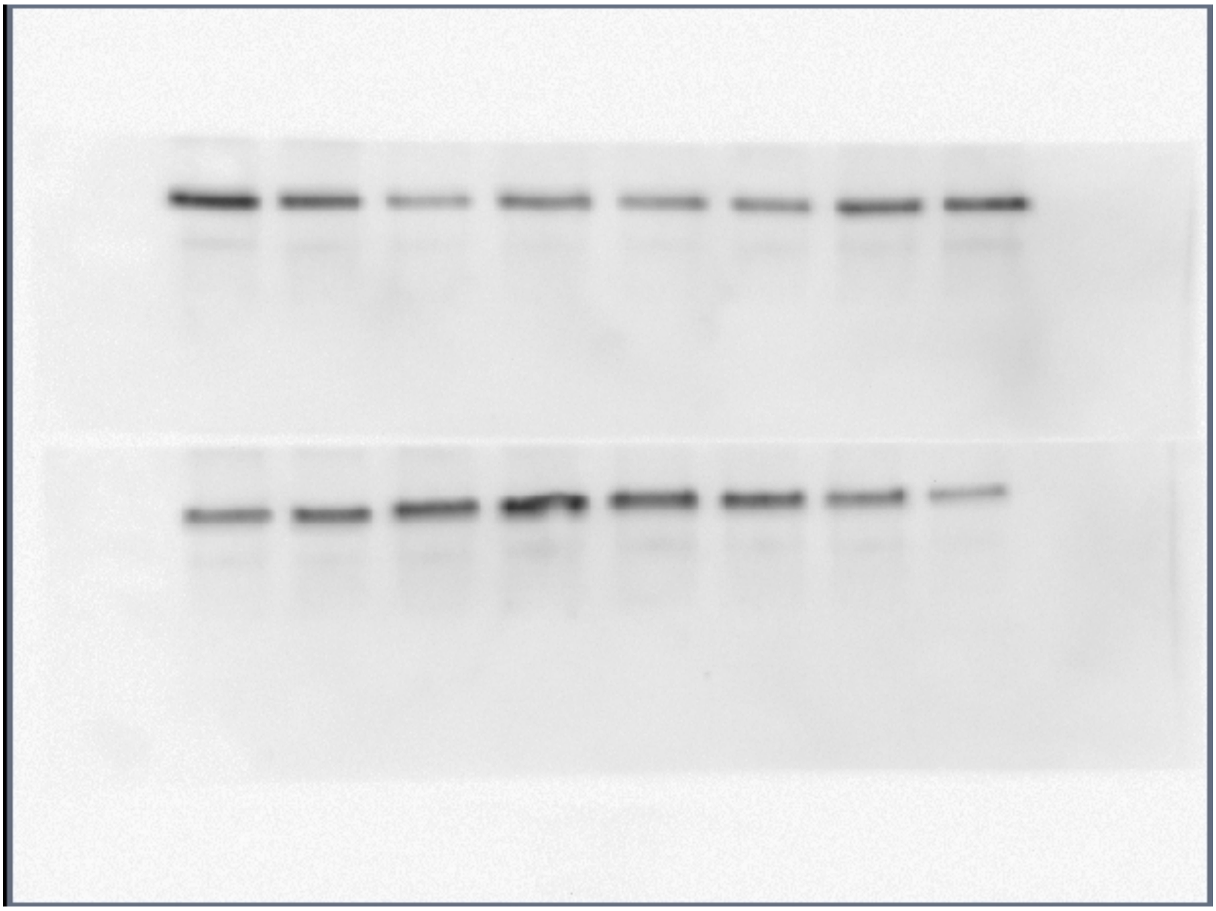

Cavβ2 (from left to right: 4 wildtype samples and 4 *Cacna1c*$^{+/-}$ samples from 8 different animals on each blot, two blots. Membranes were cut before antibody application. Upper membrane contains samples #1-4 of each genotype, lower membrane samples #5-8 of each genotype.)

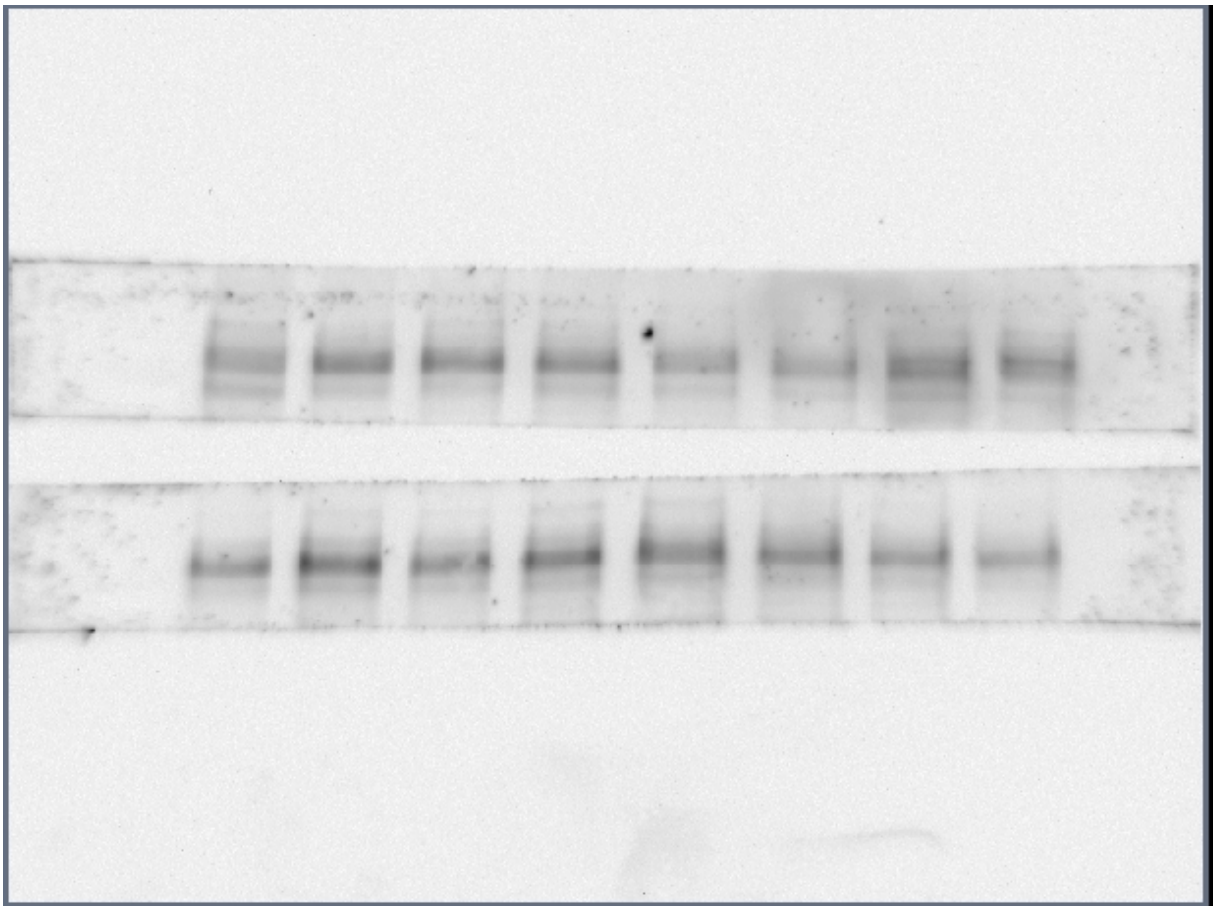

Associated GAPDH membranes (Upper membrane contains samples #1-4 of each genotype, lower membrane samples #5-8 of each genotype.)

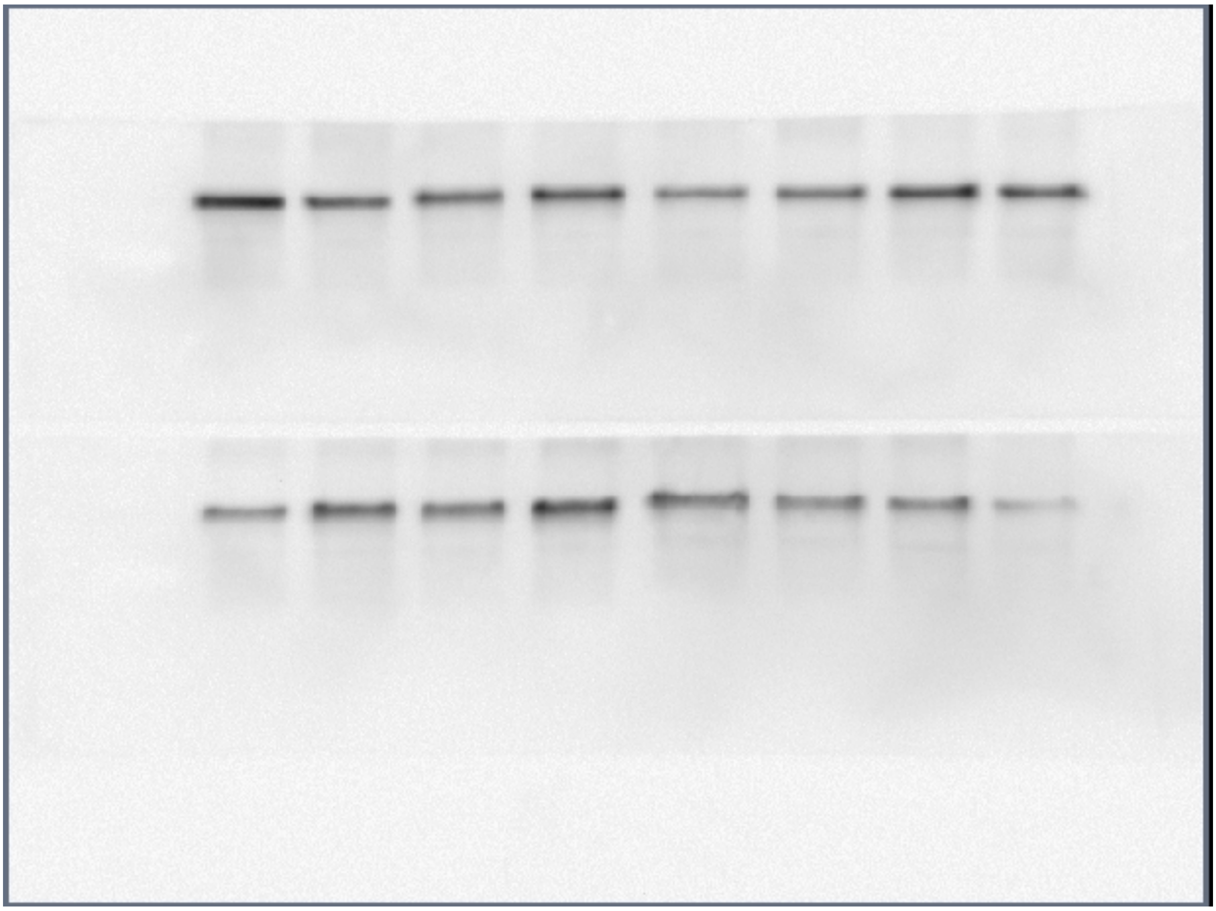

1.2 Original Western Blot images from Figure 2

Cav1.2 pS1928 (from left to right: 8 samples from Sprague-Dawley rat hearts treated with either Phosphorylation (odd lanes) or Dephosphorylation solution (even lanes).

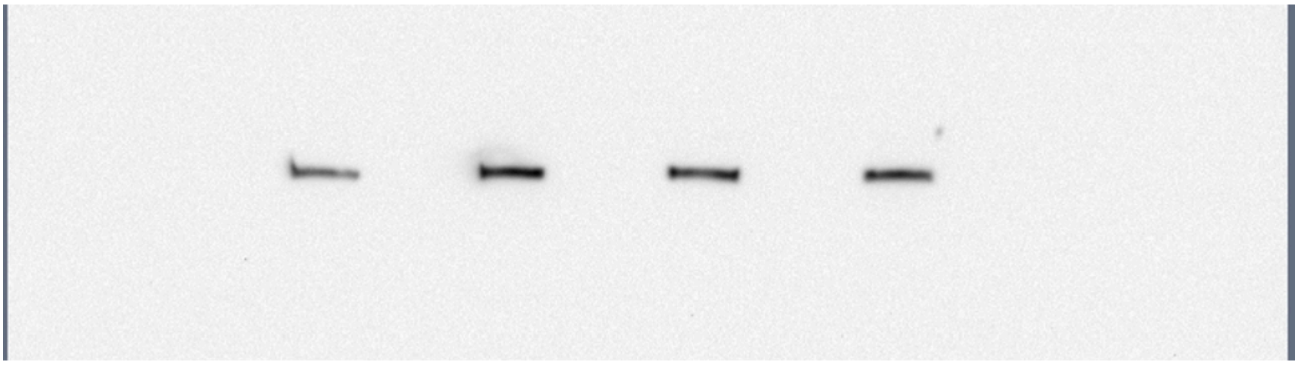

Associated GAPDH membranes.

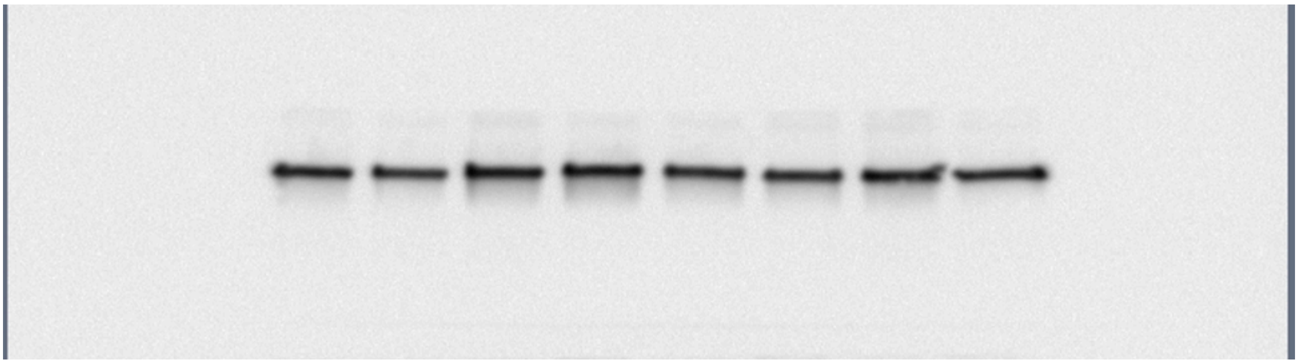

1.3 Original Western Blot images from Figure 3

Cav1.2 (from left to right: 4 wildtype samples and 4 *Cacna1c*$^{+/-}$ samples from 8 different animals on each blot, two blots. Membranes were cut before antibody application. Upper membrane contains samples #1-4 of each genotype, lower membrane samples #5-8 of each genotype.)

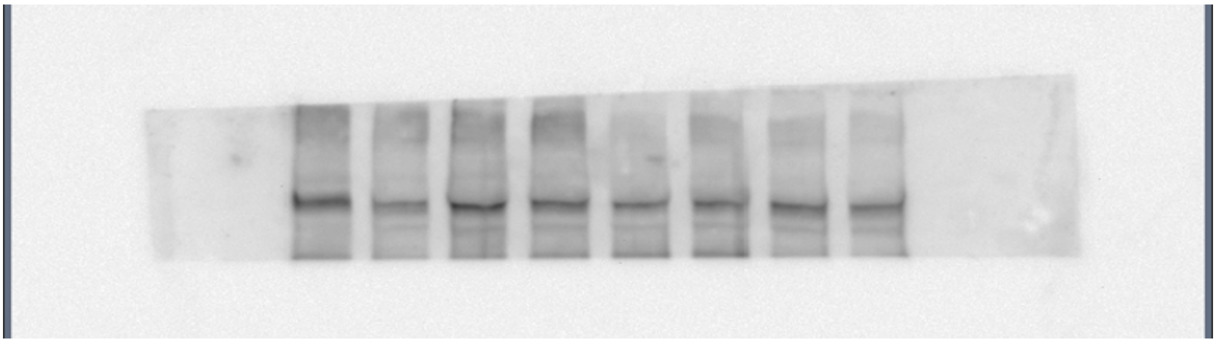

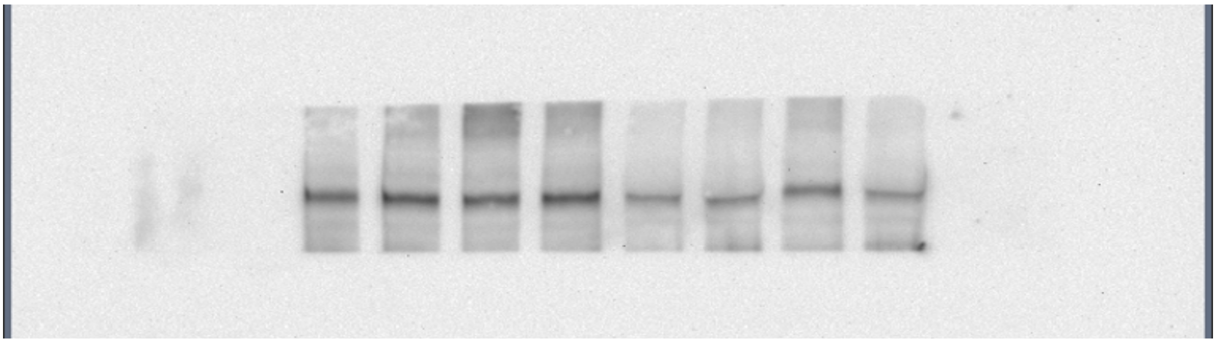

Associated GAPDH membranes (Upper membrane contains samples #1-4 of each genotype, lower membrane samples #5-8 of each genotype.)

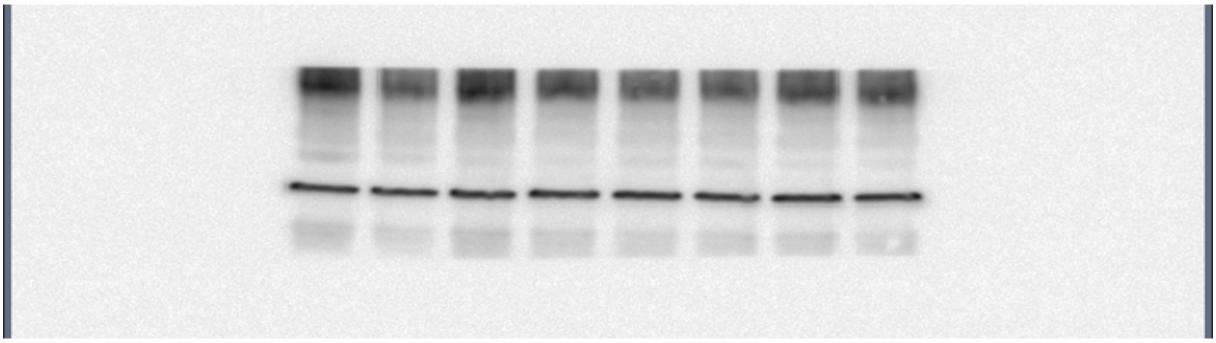

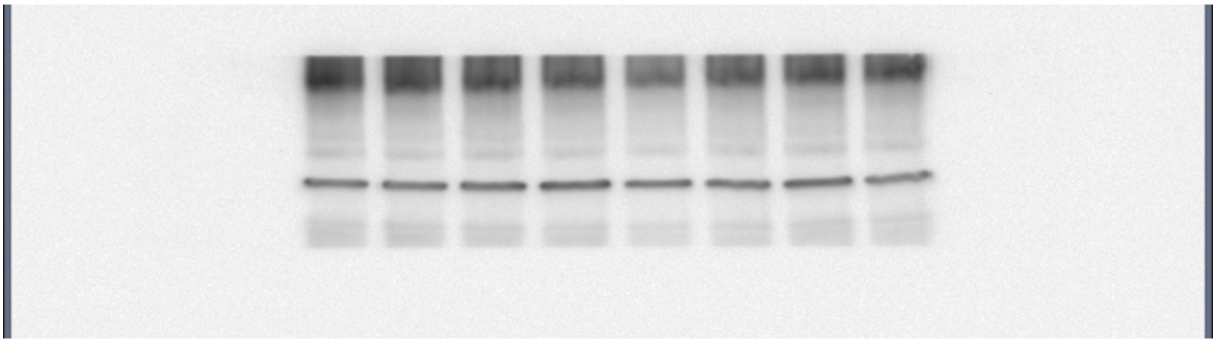

Cav1.2 pS1928 (from left to right: 4 wildtype samples and 4 *Cacna1c*$^{+/-}$ samples from 8 different animals on each blot, two blots. Membranes were cut before antibody application. Molecular weight ladder is visible on the left and right from the 8 sample lanes. Upper membrane contains samples #1-4 of each genotype, lower membrane samples #5-8 of each genotype.)

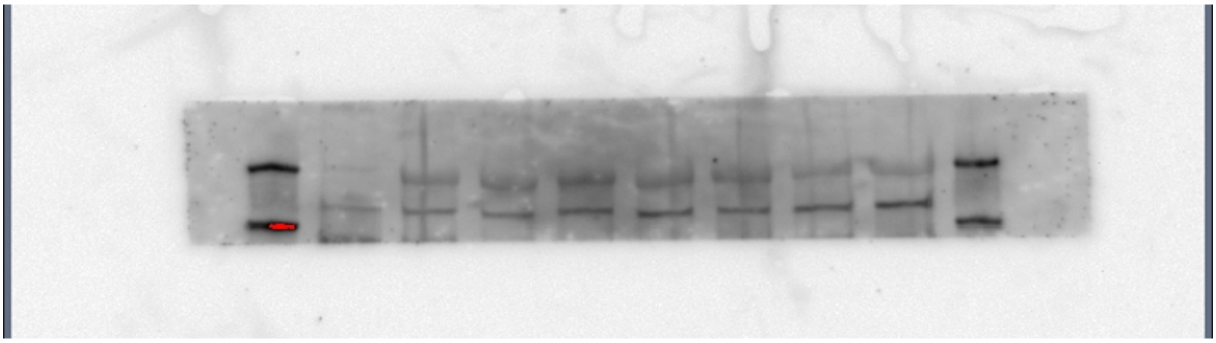

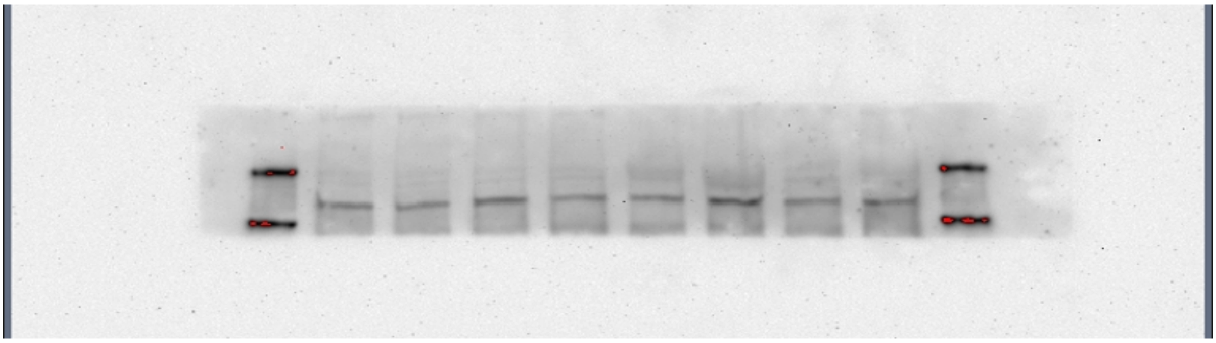

Associated GAPDH membranes (Upper membrane contains samples #1-4 of each genotype, lower membrane samples #5-8 of each genotype.)

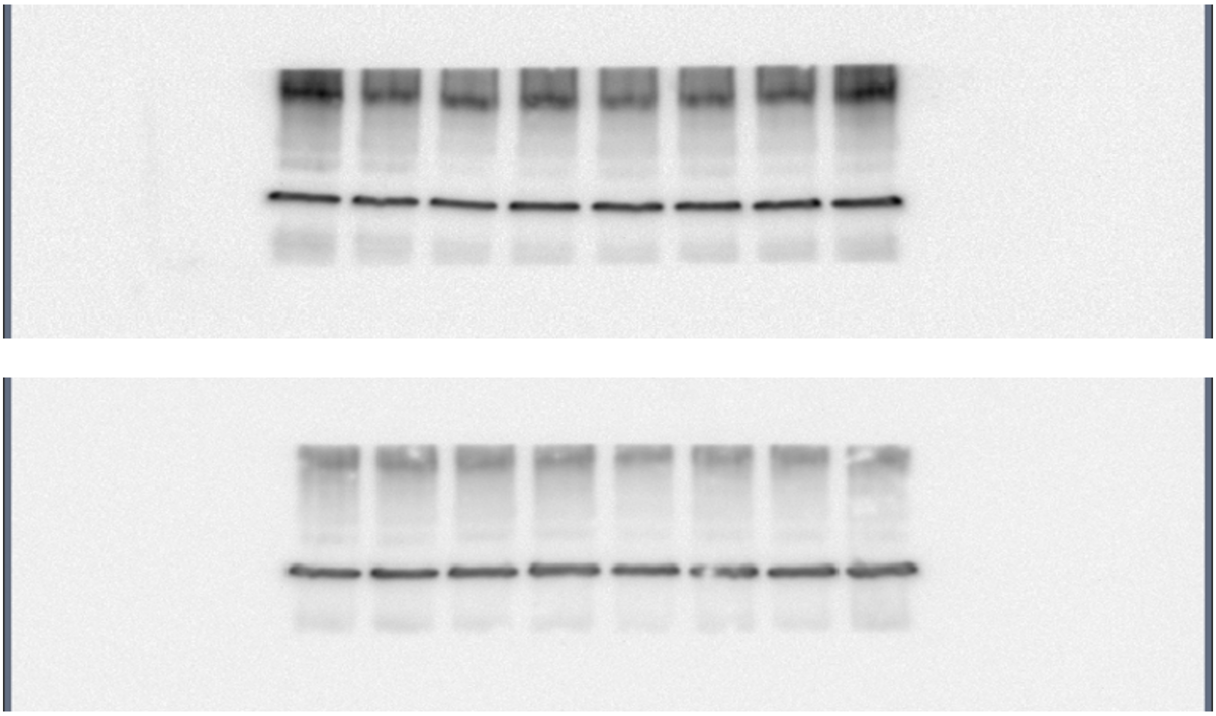

1.4 Original Western Blot images from Figure 4

Cav1.2 (from left to right: wildtype samples (lanes 1, 2, 5, 6) and *Cacna1c*$^{+/-}$ samples (lanes 3, 4, 7, 8) from 8 different animals on each blot, four blots (32 animals in total). Odd samples are controls, even samples are isoprenaline-treated. Membranes were cut before antibody application. First membrane contains samples #1-4 of each genotype, second membrane samples #5-8 of each genotype, third membrane samples #9-12 of each genotype, fourth membrane samples #13-16 of each genotype.)

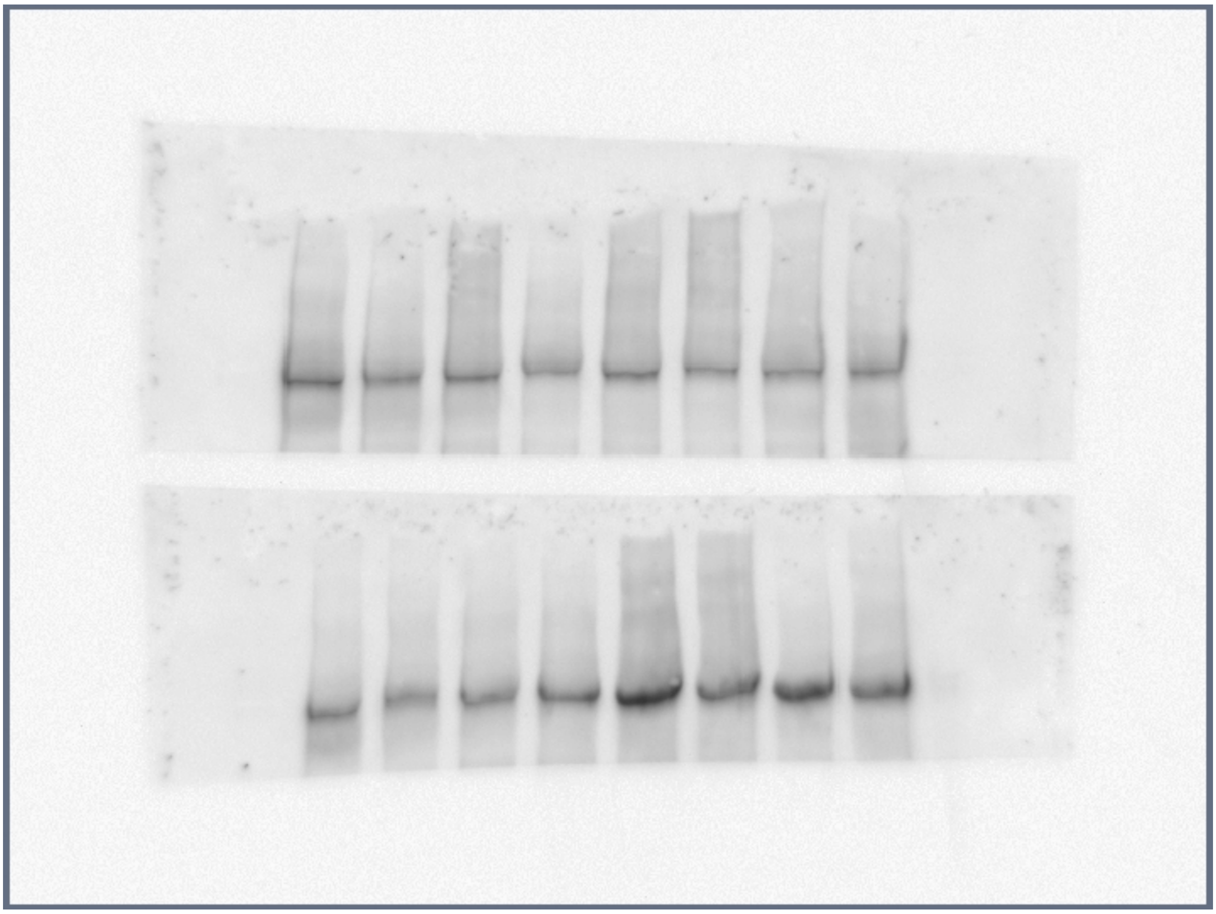

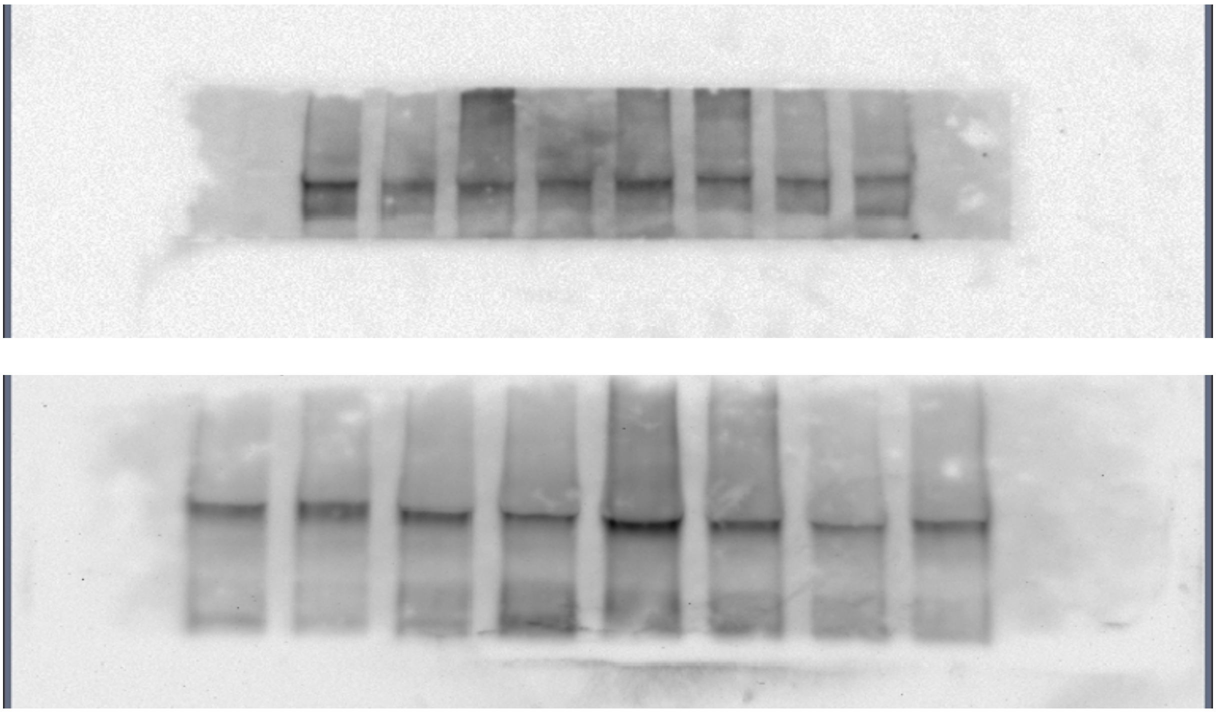

Associated GAPDH membranes (Odd samples are controls, even samples are isoprenaline-treated. Membranes were cut before antibody application. First membrane contains samples #1-4 of each genotype, second membrane samples #5-8 of each genotype, third membrane samples #9-12 of each genotype, fourth membrane samples #13-16 of each genotype.)

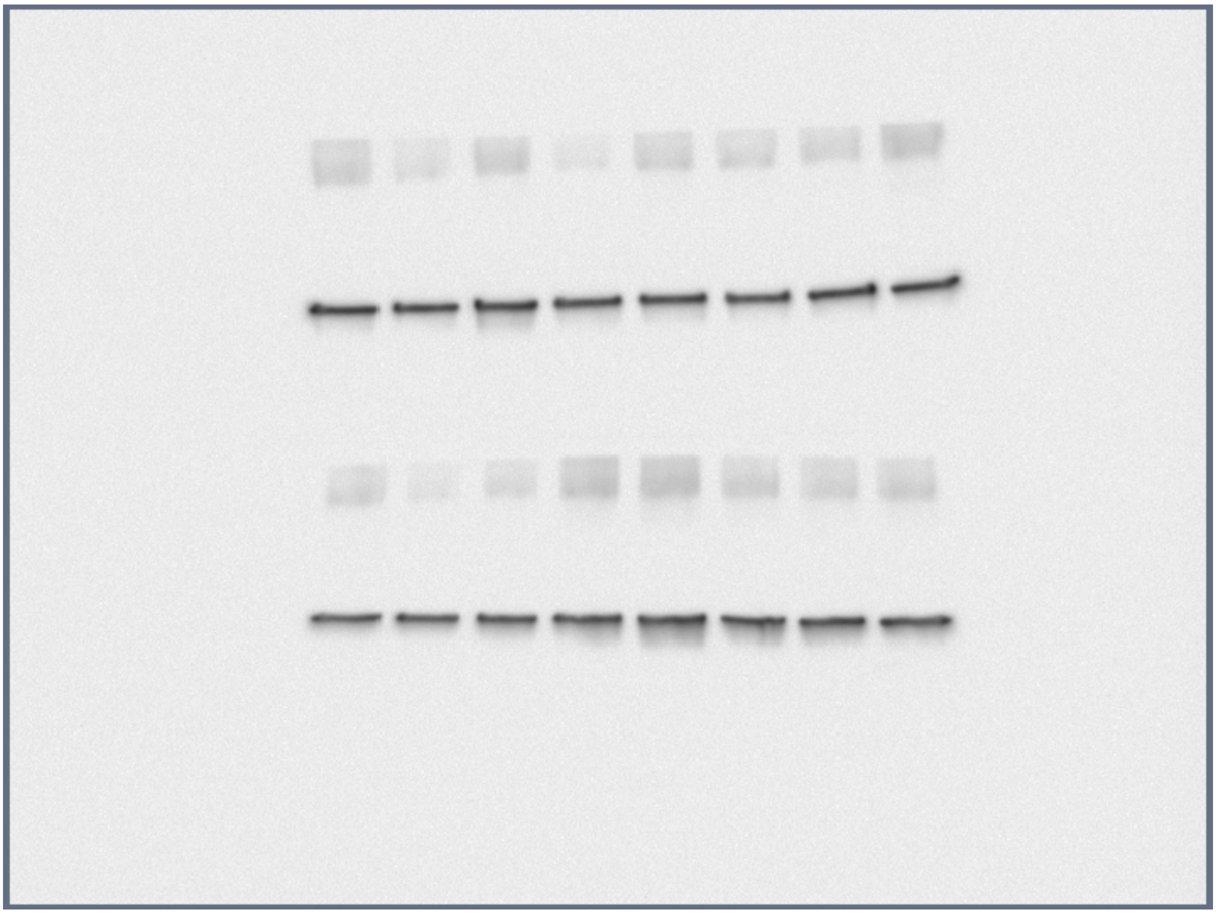

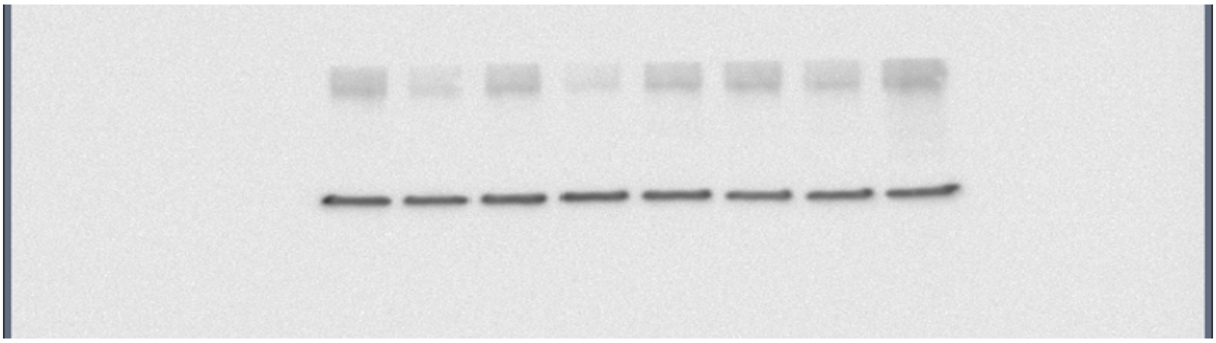

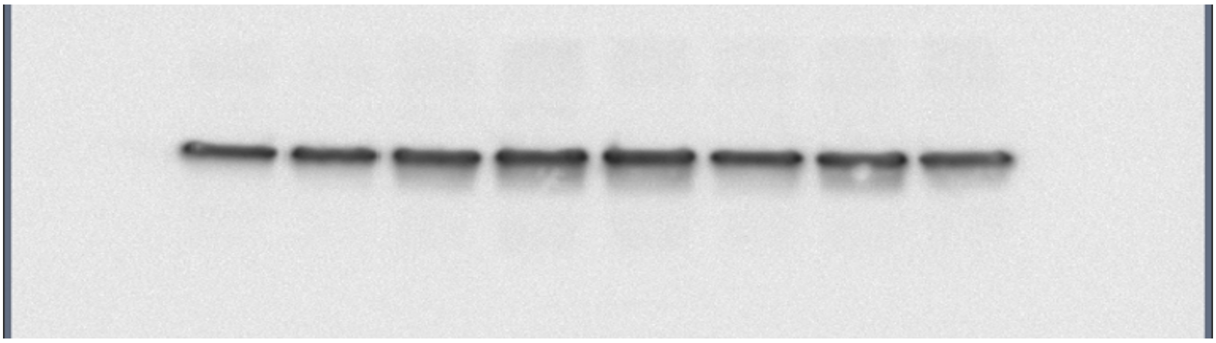

Cav1.2 pS1928 (from left to right: wildtype samples (lanes 1, 2, 5, 6) and *Cacna1c*$^{+/-}$ samples (lanes 3, 4, 7, 8) from 8 different animals on each blot, four blots (32 animals in total). Molecular weight ladder is visible on the left and right from the 8 sample lanes. Odd samples are controls, even samples are isoprenaline-treated. Membranes were cut before antibody application. First membrane contains samples #1-4 of each genotype, second membrane samples #5-8 of each genotype, third membrane samples #9-12 of each genotype, fourth membrane samples #13-16 of each genotype.)

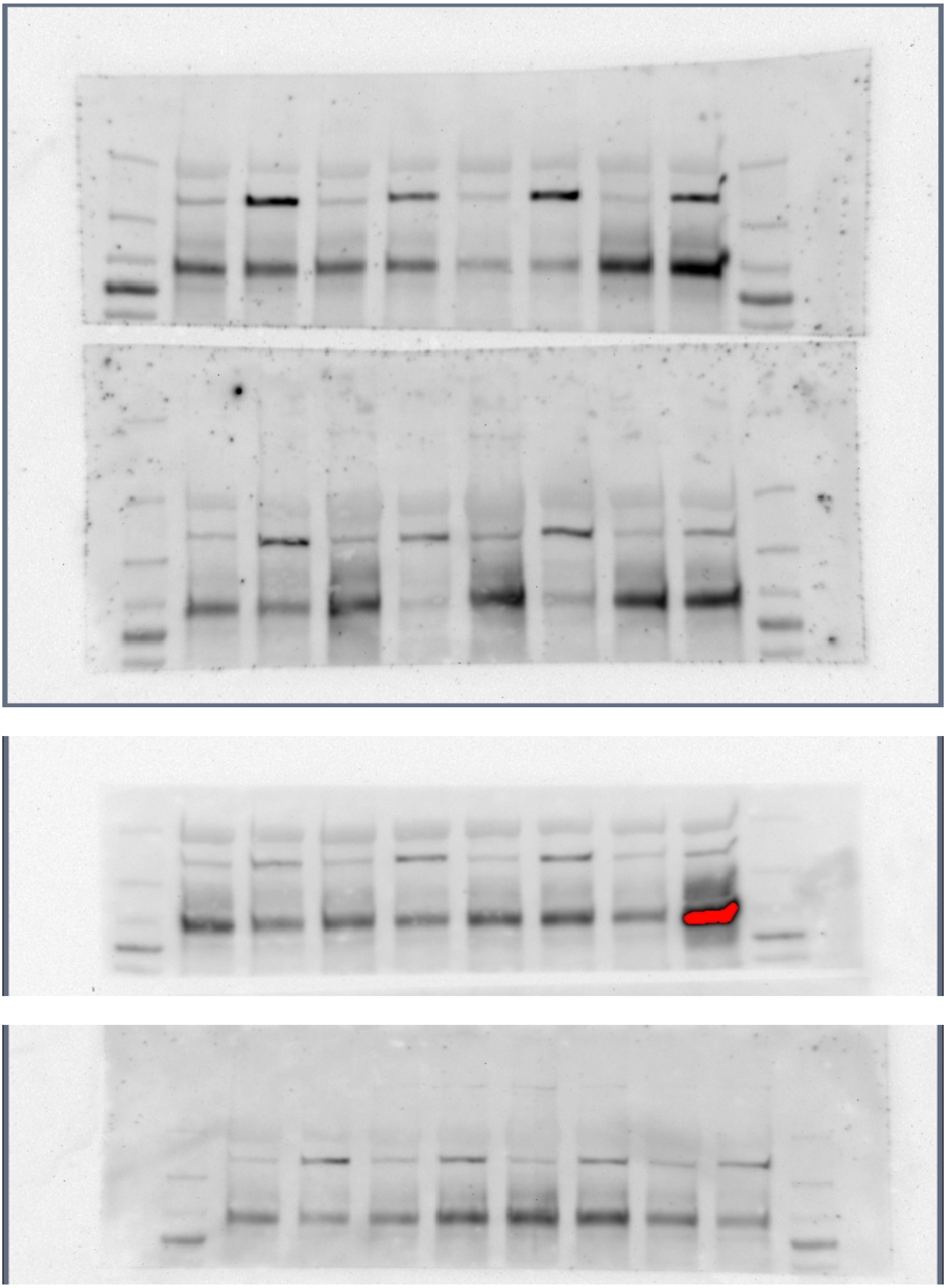

Associated GAPDH membranes (Odd samples are controls, even samples are isoprenaline-treated. Membranes were cut before antibody application. First membrane contains samples #1-4 of each genotype, second membrane samples #5-8 of each genotype, third membrane samples #9-12 of each genotype, fourth membrane samples #13-16 of each genotype.)

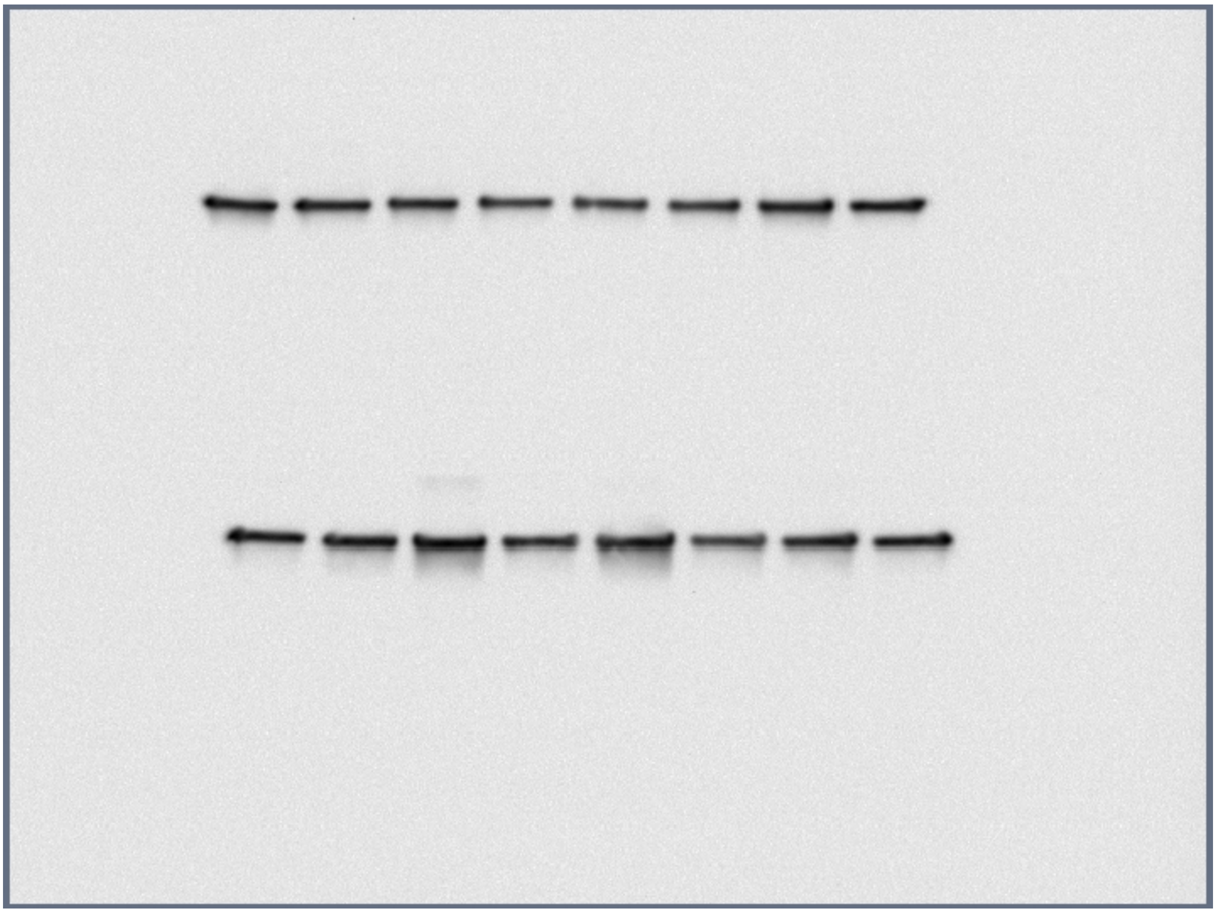

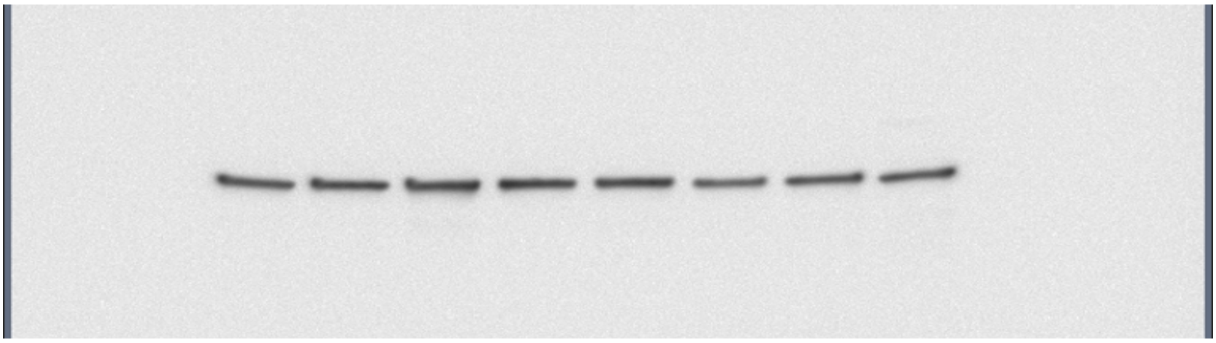

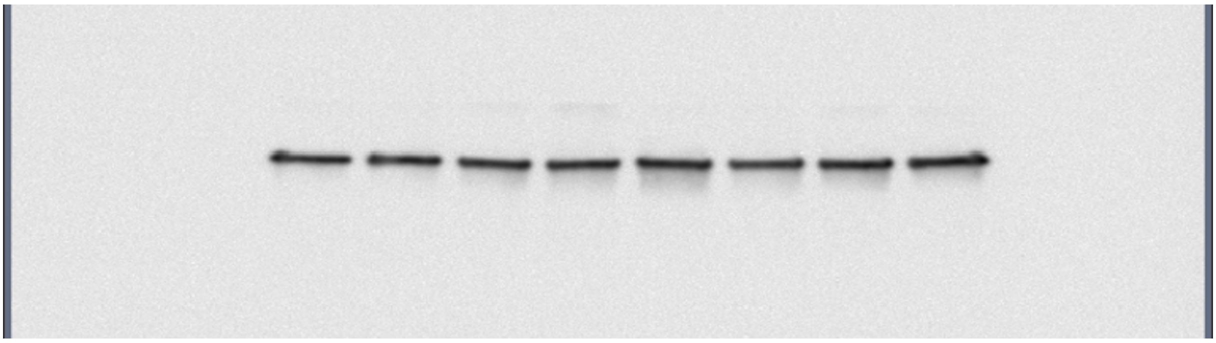

Supplement: Supplementary file 1 [file ijms-25-13713-s001.zip › ijms-3328893-supplementary.pdf]
